# Controlling the Assembly of Coiled–Coil Peptide Nanotubes

**DOI:** 10.1002/anie.201509304

**Published:** 2015-12-14

**Authors:** Franziska Thomas, Natasha C. Burgess, Andrew R. Thomson, Derek N. Woolfson

**Affiliations:** ^1^ School of ChemistryUniversity of BristolCantock's CloseBristolBS8 1TSUK; ^2^ Institute for Organic and Biomolecular ChemistryGeorg-August-Universität GöttingenTammannstrasse 237077GöttingenGermany; ^3^ Bristol Centre of Functional Materials, HH Wills Physics LaboratoryUniversity of BristolTyndall AvenueBristolBS8 1TLUK; ^4^ School of BiochemistryUniversity of BristolMedical Science Building, University WalkBristolBS8 1TDUK; ^5^ BrisSynBioUniversity of Bristol, Life Science BuildingTyndall AvenueBristolBS8 1TQUK

**Keywords:** native chemical ligation, peptide nanotubes, self-assembly, transmission electron microscopy, α-helical barrels

## Abstract

An ability to control the assembly of peptide nanotubes (PNTs) would provide biomaterials for applications in nanotechnology and synthetic biology. Recently, we presented a modular design for PNTs using α‐helical barrels with tunable internal cavities as building blocks. These first‐generation designs thicken beyond single PNTs. Herein we describe strategies for controlling this lateral association, and also for the longitudinal assembly. We show that PNT thickening is pH sensitive, and can be reversed under acidic conditions. Based on this, repulsive charge interactions are engineered into the building blocks leading to the assembly of single PNTs at neutral pH. The building blocks are modified further to produce covalently linked PNTs via native chemical ligation, rendering ca. 100 nm‐long nanotubes. Finally, we show that small molecules can be sequestered within the interior lumens of single PNTs.

Self‐assembling peptide‐based materials have become progressively more established in biomedicine and nanotechnology, with potential applications as matrices for tissue‐engineering, drug‐delivery systems, and templates for mineralization and metalation.^1^ Owing to their large internal surface areas, peptide nanotubes (PNTs) potentially expand the possible applications to filtration and storage devices, sensors, or even catalysts.^2^ To date, PNT designs have used Fmoc‐dipeptides,^3^ cyclic β‐sheet stacking peptides,^4^ lock‐washer α‐helical bundles as the building blocks,^5^ or short peptides that self‐assemble into spiral tapes.^6^ Recently, we presented a generic modular approach to assemble PNTs from α‐helical barrels (αHBs), Figure 1.^7^

**Figure 1:**
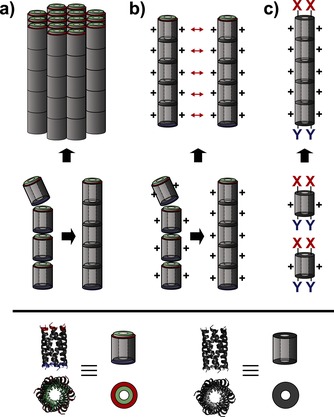
Schematic representation for PNT assembly. a) Self‐assembly of broadened PNTs based on CC‐Hex‐T; b) self‐assembly of single PNTs based on CC‐Hex‐T+; c) covalent assembly of CC‐Hex‐T+co, where X and Y represent the ligatable thiol and thioester groups (in (a) and (b) red: positively charged *N*‐terminus, blue: negatively charged *C*‐terminus). Sequences for CC‐Hex‐T, CC‐Hex‐T+, and CC‐Hex‐T+co, are given in Table 1.

The application of peptide‐based materials requires good control over self‐assembly and material properties. For PNTs this is currently best achieved by self‐organizing systems based on cyclic β‐sheet stacking peptides.^8^ In these cases, the inner diameter can be tailored via the ring size of the peptide.^2^ The modular approach that we present to assemble PNTs from αHBs allows similar control mechanisms.7a We have shown that the inner diameter varies with the oligomer state of the αHB, allowing PNTs with channels of 5–7 Å to be achieved. However, all of these PNTs assemble into broadened fibers, Figure 1 a. Controlling this broadening would represent a further step towards functional αHB‐based PNTs. Herein we describe strategies for this, which include the use of pH and sequence redesigns to make single PNTs, Figure 1 b. We compare spontaneous and covalent assembly of the PNTs, Figure 1 b,c, and, we show that different PNTs can discriminate in the encapsulation of small molecules.

Our first‐generation PNT designs use designed blunt‐ended αHBs.7c To promote end‐to‐end self‐assembly, these are permuted to expose hydrophobic patches at the *N*‐termini, and leave overall and complementary negative and positive charges at the *C*‐ and *N*‐termini, respectively, Figure 1.7a As mentioned, these redesigns associated both longitudinally and laterally to give broadened assemblies compared to the widths of the building blocks (Figure 2 a,b). To address this herein, we focus on further redesign of the hexameric building block, CC‐Hex‐T. This is well‐characterized with a 6 Å channel that is stable to certain mutations.7b, 10

Initially, we investigated the pH‐dependence of lateral assembly. We posited that fiber broadening of CC‐Hex‐based nanotubes should be pH sensitive as protonation of the glutamate (E) residues at low pH would leave the building blocks with a +30 charge from the 5 lysine residues (K) in the sequence, Table 1. As predicted, at low pH thin fibers were observed (Figure 2). Moreover, fibrils consistent with single PNTs (ca. 3–4 nm, Table S1 in the Supporting Information) were observed by negative‐stain transmission electron microscopy (TEM) below pH 5.6 (Figure 2 c). Circular dichroism (CD) spectroscopy confirmed this in solution. Owing to chiral scattering, broadened fibrous α‐helical systems, such as CC‐Hex‐T at pH 7.4, give red‐shifted CD spectra of reduced intensity, Figure 2 b, compared with typical α‐helical spectra.^11^ However, decreasing the pH for CC‐Hex‐T samples gave increased signal and loss of the red shift, with the transition complete by pH 5.6 (Figure 2 d). This disassembly of the fibers, but not of the α‐helical structure, in acidic conditions was reversible and thickened fibers returned upon increasing pH (Figure S2).

**Figure 2:**
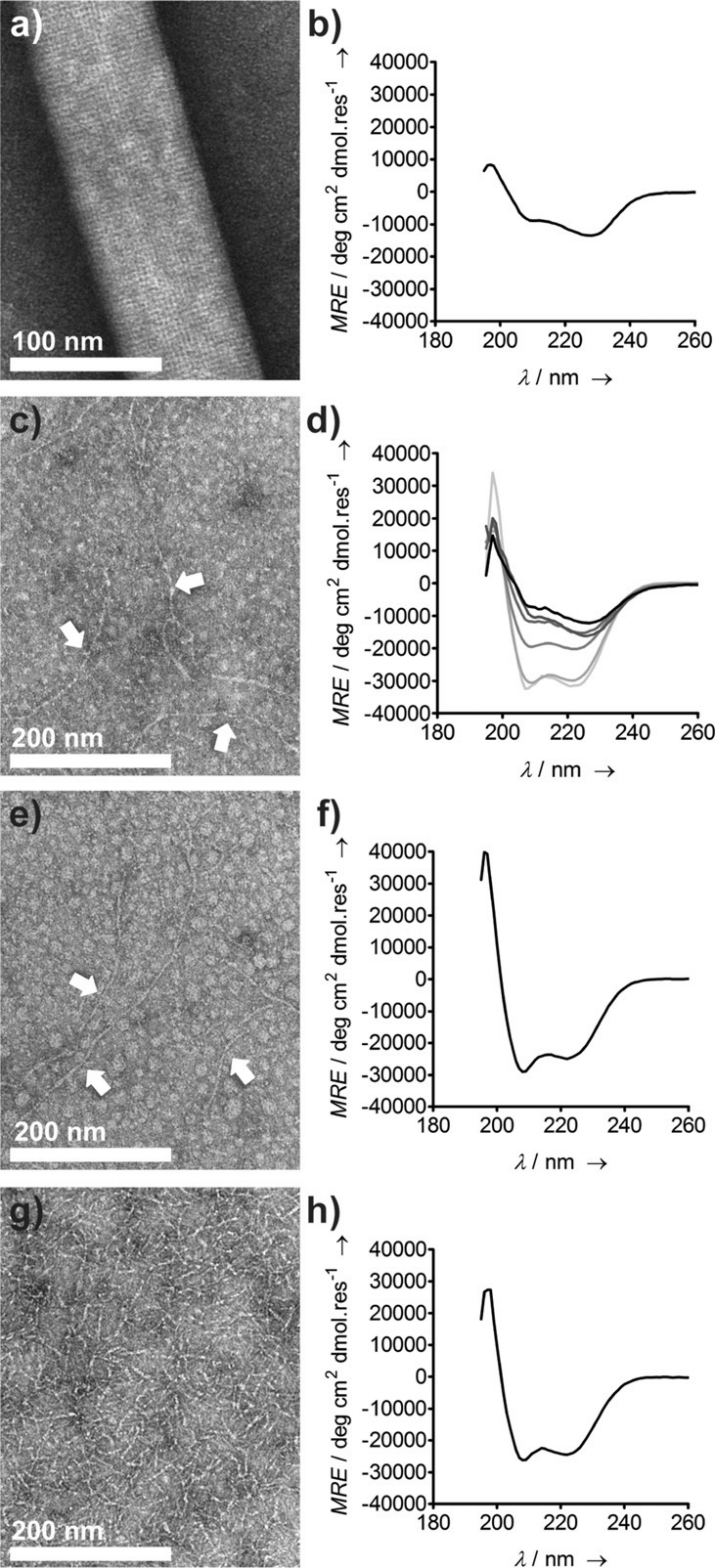
Characterization of PNTs formed by hexameric building blocks. Negative‐stain TEM images of a) CC‐Hex‐T; c) CC‐Hex‐T at pH 5.0; e) CC‐Hex‐T+; and g) CC‐Hex‐T+co after 7 day equilibration at 25 °C. The white arrows in (c) and (e) point out thin fibrils (single PNTs) that are otherwise difficult to see. CD spectra for b) CC‐Hex‐T; d) CC‐Hex‐T at pH 7.4, 6.7, 6.0, 5.6, 5.1, and 3.1 (black‐to‐gray gradient: from pH 7.4 (black) to pH 3.1 (light gray)); f) CC‐Hex‐T+; and h) CC‐Hex‐T+co monomer building block.^9^

**Table 1:** Sequences of designed PNT‐forming peptides.

Register	***abcdefg***	***abcdefg***	***abcdefg***	***abcdefg***
CC‐Hex‐T^[a]^	H‐LKAIAQE	LKAIAKE	LKAIAWE	LKAIAQE‐OH
CC‐Hex‐T+^[a]^	H‐LKAIAKE	LKAIAKE	LKAIAWE	LKAIAKE‐OH
CC‐Hex‐T+co^[a]^	H‐CKAIAKE	LKAIAYE	LKAIAKE	LKAIAKQ‐SBzl

[a] The nomenclature is based on the oligomeric state of the monomer building block, which is a coiled–coil hexamer.

The observation of reduced fiber thickening at low pH suggested a redesign of CC‐Hex‐T to make single PNTs at neutral pH. We reasoned that increasing the positive charge on the outer surfaces of the fibrils should prevent bundling to form fibers, Figure 1 b. In coiled‐coil structures, the ***f*** positions of the underlying sequence repeat, ***abcdefg*** (Table 1), fall on this outer surface.^12^ For CC‐Hex‐T+, we mutated all but one of these to K giving an overall positive charge of +3 per peptide, and +18 per CC‐Hex building block, at neutral pH, Table 1.

When equilibrated at pH 7.4, CC‐Hex‐T+ showed exclusively extended fibrils up to about 1 micron in length in TEM, with diameters of around 3–4 nm (Figures 2 e and Table S2), that is, consistent with single PNTs; and without chiral scattering in the CD spectra, Figure 2 f.

To improve stability of the single PNT fibrils, we attempted to link the CC‐Hex‐T building blocks covalently through native chemical ligation (NCL), Figure 1 c.^13^ To enable this, we made a third peptide, CC‐Hex‐T+co, in which CC‐Hex‐T+ was modified to include an *N*‐terminal cysteine (C) and a *C*‐terminal thioester (Table 1, Scheme 1).

**Scheme 1:**
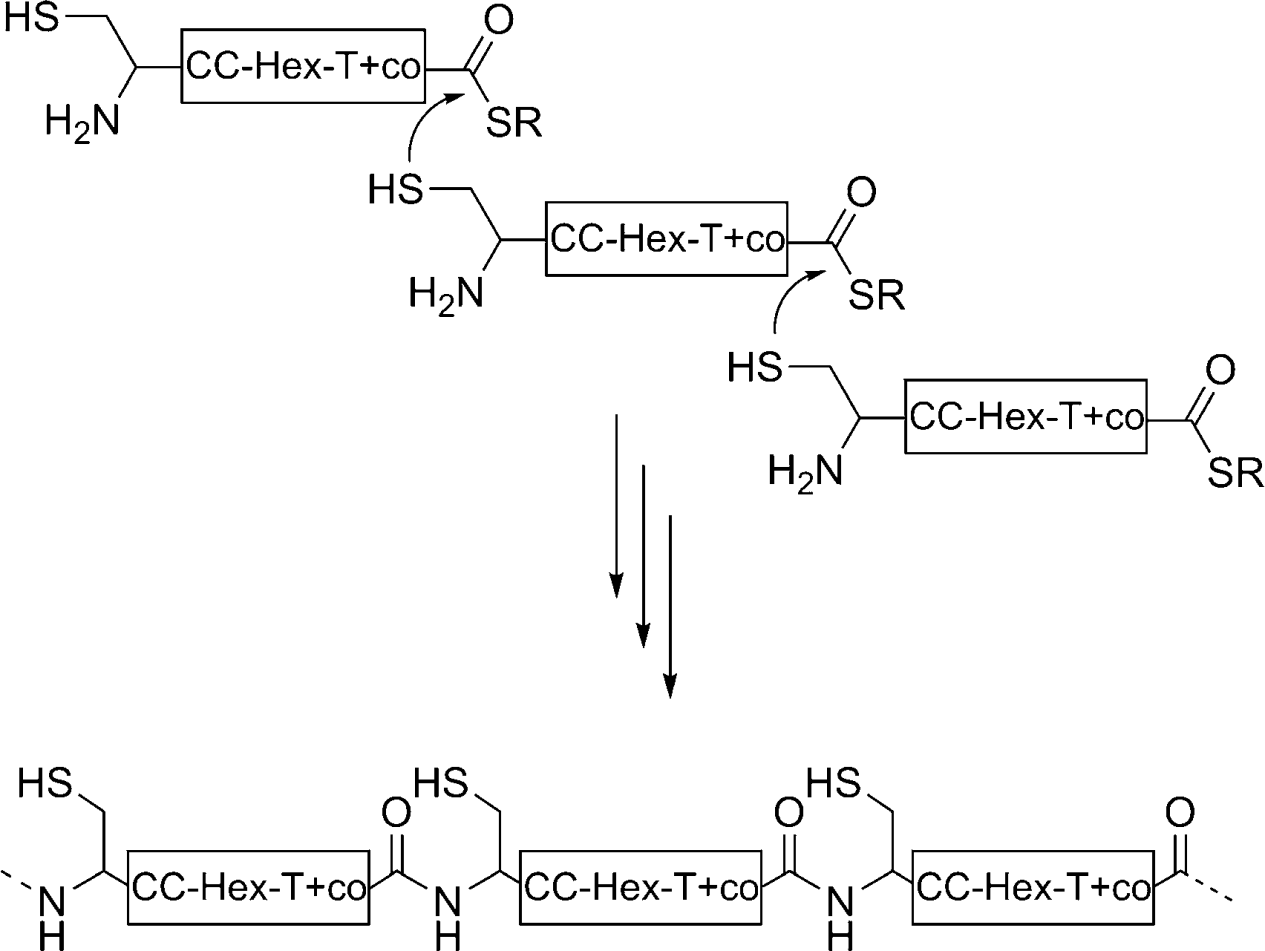
Oligomerization of CC‐Hex‐T+co, by native chemical ligation (NCL).

CD spectra before NCL showed stably folded α‐helices (Figure 2 h).^10^ Thus, we assume that polymerization occurs mostly via assembled CC‐Hex‐based building blocks. After 1 day of reaction, high‐molecular‐weight species appeared in SDS‐PAGE (Figure S4 c), and TEM revealed approximately 100 nm long fibrils (Figure 2 g).

To explore the timeframe of oligomerization, we monitored the reaction by high‐pressure liquid chromatography (HPLC), SDS‐PAGE, TEM, and mass spectrometry (MS). HPLC showed over 80 % conversion of the starting material after 5 h (Figure S5). This was confirmed by SDS‐PAGE (Figure 3 g): after 1 day, the monomer band was faint; the appearance and disappearance of a band consistent with dimeric non‐self‐assembling CC‐Hex‐T+co was visible (Figure S6); and a high‐molecular‐weight smear formed over time. MS revealed dimers to octamers after three hours (Figures 3 h and Figure S7). However, small amounts of dimeric and trimeric cyclic species were also present. Corroborating this, TEM images revealed short fibrils of 30–40 nm in length after 30 min, with the average fiber length increasing up to 100 nm after 7 days (Figure 3 a–f, Figure S8,S9). We suggest that the observation of a limiting length is due to many nucleation sites, and the precipitation of the covalent PNTs from solution.

**Figure 3:**
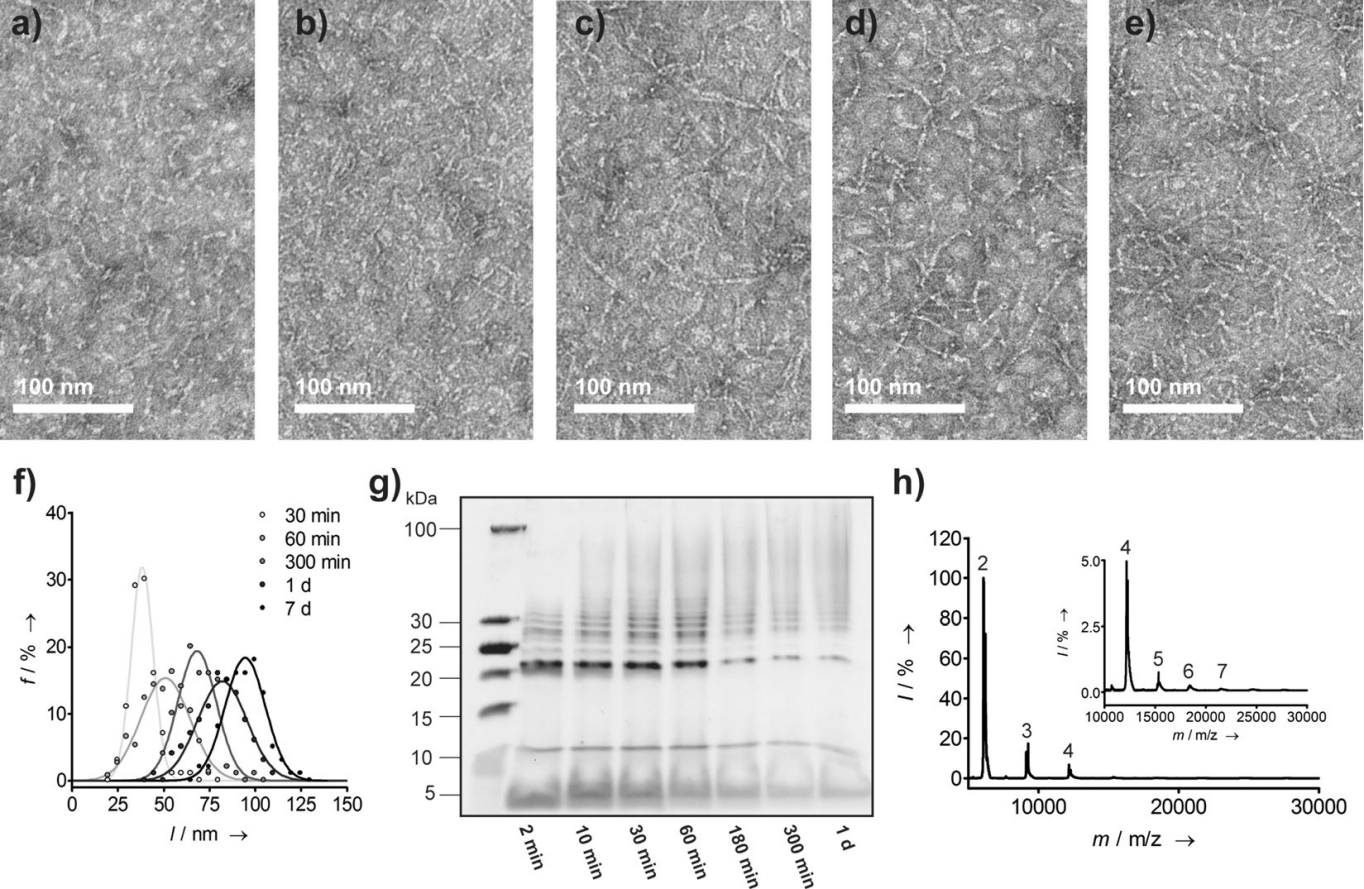
PNT formation by NCL over time. a–e) Negative stain TEM at different times: a) 30 min; b) 60 min; c) 300 min; d) 1 day; and e) 7 days. f) Corresponding distribution of fiber lengths with time.^9^ g) SDS‐PAGE of oligomerization reaction sample at different times, h) Maldi‐TOF mass spectrum of the oligomerization reaction after 3 h (numbers signify the oligomeric species).

We turned to linear dichroism (LD) spectroscopy to verify the secondary and quaternary structure of the PNTs in solution.14a This requires the alignment of molecules, usually by shear flow, and only gives signal for those with large aspect ratios. We observed LD signals for both heat‐treated CC‐Hex‐T at low pH—which gave longer fibrils than untreated samples, Figure S9—and CC‐Hex‐T+ at neutral pH, Figure 4 a,b. The resulting spectra indicated aligned α‐helical rods with the helices parallel to the long axis of the rods.^14^ Moreover, these correlated with fibril lengths observed in TEM: the longer CC‐Hex‐T+ fibrils gave the more intense LD signal, and required lower alignment forces, Figure 4 b, than the heated CC‐Hex‐T fibrils, Figure 4 a. In contrast, any LD signals for spontaneously assembled CC‐Hex‐T, and for covalently linked CC‐Hex‐T+co were too weak to be observed; presumably, this reflects relatively short fibers formed by these systems, which were ≤100 nm and at the limit of the size required for LD spectroscopy, Figure S8–S10.^14^

**Figure 4:**
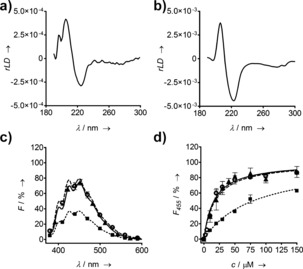
Spectroscopic characterization of the PNTs. a),b) LD spectra for a) CC‐Hex‐T heated and then cooled at pH 5.1, recorded at 6030 RPM; and b) CC‐Hex‐T+ at pH 7.4, 1260 RPM. c),d) Binding of DPH into the channels of CC‐Hex‐T (triangles, solid line), CC‐Hex‐T+ (squares, dotted line), and CC‐Hex‐T+co (circles, dashed line). c) Fluorescence spectra, and d) saturation binding curves recorded at 455 nm.

Finally, to probe the accessibilities of the inner channels of the single PNTs, and to assess their utility as components of sequestration, storage, and delivery devices, we tested the encapsulation of the small hydrophobic dye 1,6‐diphenylhexatriene (DPH). In hydrophobic environments, DPH fluoresces, Figure 4 c, but this is quenched in water. DPH binds strongly to thickened CC‐Hex‐T PNTs (Figure 4 d).7a Although DPH bound to both of the single‐PNT redesigns, Figure 4 c, the saturation binding curve for CC‐Hex‐T+ showed reduced binding affinity compared to CC‐Hex‐T, Figure 4 d. We propose that this is best explained by CC‐Hex‐T+ PNTs being less stable than CC‐Hex‐T, as any stabilization from lateral association of fibrils will be lost in the CC‐Hex‐T+ PNTs. In turn, this facilitates release of bound DPH. Supporting this idea, the covalently linked PNTs of CC‐Hex‐T+co showed binding behavior similar to CC‐Hex‐T, Figure 4 c,d. We suggest that covalent linkage of the monomers into the extended tubular structures prevents PNT disassembly, and, therefore, increases the binding affinity to small hydrophobic molecules.

In summary, we have demonstrated alternative strategies to control the lateral and longitudinal assembly of α‐helical PNTs. These systems range from non‐covalent assemblies—namely, stable thickened PNTs, which unbundle under acidic conditions; and more‐dynamic single PNTs achieved through rational redesign—to highly stable covalently linked single PNTs. The different assembly modes alter both the morphologies of the PNTs, and their properties, including the uptake and release of hydrophobic molecules. In future, this could guide the design of PNT‐based delivery systems or storage devices.

There are a small number of other PNT systems that display elements of control over assembly that we demonstrate herein. These include cyclic β‐structured peptides that stack,^8^ hydrophobic dipeptide‐based PNTs,^15^ and, in part, the self‐assembling spiral tapes based on disulfide‐linked octapeptides.^16^ Our designs carry certain advantages, principally modularity and generality. Therefore, the control mechanisms that we describe should be readily transferrable to other αHB building blocks of varying pore size.7a,7c The inner lumens of these barrels are also mutable,7b, 10 which should facilitate further the design of functional PNTs.

## Supporting information

As a service to our authors and readers, this journal provides supporting information supplied by the authors. Such materials are peer reviewed and may be re‐organized for online delivery, but are not copy‐edited or typeset. Technical support issues arising from supporting information (other than missing files) should be addressed to the authors.

Supplementary
