# Supplementary material for: Controlling the Assembly of Coiled–Coil Peptide Nanotubes
Source: Angew Chem Int Ed Engl. 2015 Dec 14;55(3):987–91. doi: 10.1002/anie.201509304 (PMC4744968; doi:10.1002/anie.201509304)
Supplement: Supplementary file 1 — Supplementary [file ANIE-55-987-s001.pdf]

Angewandte Chemie
Eine Zeitschrift der Gesellschaft Deutscher Chemiker

Supporting Information

Controlling the Assembly of Coiled–Coil Peptide Nanotubes

*Franziska Thomas, Natasha C. Burgess, Andrew R. Thomson, and Derek N. Woolfson**

anie_201509304_sm_miscellaneous_information.pdf

Table of Contents

Materials and Methods 2
Additional Figures and Tables 4
References...12

Materials and Methods

General. Fmoc-protected amino acids, HBTU and peptide grade DMF were purchased from *AGTC Bioproducts* (Hessle, U.K.). The H-Glu HMPB-Chemmatrix® and H-Rink Amide-Chemmatrix® resin was acquired from *PCAS BioMatrix Inc.* (Saint-Jean-sur-Richelieu, Canada). All other chemicals were purchased form *Fisher Scientific* (Loughborough, U.K.). Water was purified with a *Synergy®* UV water purification system from Millipore. Unless otherwise stated, biophysical measurements were performed in phosphate buffered saline (PBS, 8.2 mM Na_2HPO_4, 1.8 mM K_2HPO_4, 137 mM NaCl, 2.7 mM KCl, pH 7.4). Peptide concentrations were determined by UV-absorbance (ε_{280} (Trp) = 5690 mol^{-1} cm^{-1}, ε_{280} (Tyr) = 1280 mol^{-1} cm^{-1}) using a *NanoDrop 2000* Spectrophotometer from *Thermo Scientific*.

Peptide Synthesis. The peptide acids were synthesized on a H-Glu HMPB Chemmatrix® resin on a 0.1 mmol scale on a *CEM* microwave-assisted synthesizer. The synthesis was conducted *via* a standard Fmoc-protocol using HBTU as coupling reagent. N-acetylation of the peptides was carried out by using acetic acid anhydride / pyridine (1:9). Acidic cleavage from the resin was achieved by a treatment of the resin with a mixture of trifluoroacetic acid (TFA) / triisopropylsilane / water (90:5:5, 3 h). The resin was extracted with additional TFA (5 mL), and the combined extracts were concentrated to a third of the initial volume under a flow of nitrogen. The crude peptide was then precipitated in cold diethylether (40 mL) and isolated by centrifugation and decantation of the ether. The precipitate was redissolved in 5 mL of a 1:1 mixture of acetonitrile and water and then freeze-dried to give a fine white solid.

The cysteinyl peptide benzyl thioester was synthesized following a side-chain anchoring strategy described by Ficht et al. Fmoc-Glu-OAll was linked to H-Rink Amide-Chemmatrix® resin.[1] The peptide chain elongation was conducted via a standard Fmoc-protocol. The N-terminal cysteine residue was introduced as a Boc-Cys(Trt)-OH building block. After peptide chain assembly the C-terminal allyl ester was hydrolyzed using $Pd[PPh_3]_4$ (0.5 eq) and $PhSiH_3$ (40 eq) for 2x 30 min reaction time. Afterwards, on-resin thioesterification was performed with benzyl mercaptane (24 eq), diisopropylcarbodiimide (30 eq), N-hydroxybenzotriazole (30 eq), and diisopropyl ethyl amine (36 eq) in dichloromethane/DMF (4:1) for 2x 5 h. Acidic cleavage was carried out as described above. The N-acetylated peptide thioester which was used to generate the reference peptide (CC-Hex-T+co)2 was synthesized accordingly.

Peptide purification. Peptides were purified by reverse-phase HPLC using a *JASCO* chromatography system and a *Kromatek* C18HQsil column (150 by 10 mm) using a linear gradient of water and acetonitrile (buffer A: water, 0.1 % TFA, buffer B: acetonitrile, 0.1 % TFA) run from 30-60 % buffer B over 30 min.

Peptide characterization. The peptides were characterized by mass spectrometry on a *Bruker Daltonics UltrafleXtreme* MALDI-TOF mass spectrometer operating in positive-ion reflector mode. (matrix: α-cyano-4-hydroxycinnamic acid (CHCA), external calibration). Analytical HPLC measurements were performed using a *JASCO* chromatography system and a *Phenomenex® Prodigy ODS-3* (5 μm, 4.6 x 100 mm). For peptide characterization a linear gradient of water and acetonitrile (buffer A: water, 0.1 % TFA, buffer B: acetonitrile, 0.1 % TFA) run from 20-80 % over 20 min was used. Chromatograms were monitored at 220 and 280 nm wavelengths.

Oligomerization by native chemical ligation. The bifunctional CC-Hex-T+co building blocks were equilibrated in degassed PBS buffer to which was added 20 mM TCEP and 50 mM ascorbic acid at 25 °C.[2] To improve the data quality in subsequent biophysical measurements the reaction solutions were dialyzed against sole PBS (CD spectroscopy and LD spectroscopy) or MOPS (10 mM MOPS, 150 mM NaCl, pH 7.2, TEM). For reaction monitoring by MALDI-TOF MS the reaction solutions were desalted prior to the measurements using C18 ZipTip® pipette tips from *Millipore*.

Circular Dichroism and Linear Dichroism. CD spectra, CD thermal-denaturation curves and LD spectra were recorded using a *JASCO J-810* spectropolarimeter fitted with a Peltier temperature

controller. CD spectra were measured at 100 μM total peptide concentration in PBS at 20 °C in 1 mm quartz cuvettes from STARNA at 50 nm / min scanning speed. CD thermal-denaturation experiments were performed by heating from 5 to 95 °C at a heat rate of 40 °C / h. The CD signal at 222 nm was recorded at 1 °C intervals (1 nm interval, 1 nm bandwidth, 16 s response time). The midpoints of the thermal denaturation curves (T_m) were determined from the second derivative of the variable temperature slope. The solution-phase CD spectra of the broadened PNTs had attenuated signals at 208 nm and red-shifted bands at 222 nm. These features indicated chiral scattering from large assemblies with dimensions comparable to the wavelength of the incident light, and, therefore, complicated interpretation of the data. CD spectra were plotted from wavelength 195 - 260 nm. The scattering associated with fibre formation also caused difficulties and introduced errors in verification of sample concentrations, complicating the calculations of MREs and % helicities usually presented for CD data.

LD spectra were measured using a micro-volume Couette flow LD cell purchased from Kromatek LTD. Samples of 400 μM peptide concentration in PBS were spun at 1260 RPM and 6030 RPM (as stated in the text) for 60 minutes before a scan was taken. LD signal was measured at standard sensitivity using 1 nm interval, 1 nm bandwidth and 200 nm/min scan speed. Signal is reported as reduced LD (rLD), where the LD signal is divided by the isotropic absorption of the stationary sample and the rLD of the stationary sample is subtracted. Spectra are presented with a post-processing moving average smoothing with a span of 3 applied. Peptide fibre samples were made at the required concentration from freeze-dried pure peptide aliquots, left to mature for 5 minutes and then 70 μL transferred to the couette for 25 minutes before the stationary spectrum was taken prior to spinning.

Negative-stain TEM. Samples were spread on continuous carbon grids and negatively stained briefly with 1% uranyl acetate. Images were acquired on a *JEOL JEM 1400* (tungsten filament) or a *JEOL JEM 1200* (tungsten filament) operating at 120 kV. Negative-stain TEM images are presented without image processing other than cropping, rotating and increasing the contrast where stated.

Fibre lengths and widths were measured from 100 fibres using *ImageJ*,[3] from EM images with a minimum magnification of 1 pixel/nm, in order to reduce measurement error. Fibre lengths were sorted into bin sizes of 5 nm, giving histograms to show the spread of sizes. Gaussian distributions are plotted and quoted for all data sets. For the fibre width we used bin sizes of 0.5 nm and calculated the mean value and the standard deviation.

SDS Page. SDS page gel electrophoreses was performed on acryl amide gels (12 % acryl amide, 10 % SDS and a Tris glycine running buffer (25 mM Tris base, 192 mM glycine, 1 % SDS) at 40 A and 30 min run time. Gels were casted manually using a recipe described on http://www.cytographica.com/lab/acryl2.html.

DPH-binding experiments. Binding experiments were carried out in a well-plate format in PBS buffer at 25 °C, and at a steady DPH concentration of 1 μM. Peptide concentrations varied from 10 to 150 μM in order to record saturation binding curves. The mixtures of peptides and DPH were left to equilibrate for 2 h at 25 °C and under gentle shaking. Fluorescence spectra were then recorded in a range of 380-600 nm with λex = 350 nm using a *Clariostar* plate reader from *BMG Labtech*. Saturation binding curves were generated from DPH fluorescence at 455 nm and fitted to a hyperbolic function to facilitate qualitative comparison. Data were normalized to Ymax (maximum Y value).The error bars in main text (Fig. 4d) are standard deviations of four measurements, and the data point represents the mean average. Curve fitting was performed using a single-site-binding model (Eq. S1). The tubular assemblies most likely exhibit multiple binding sites, hence, binding mechanisms are too complex to be captured and quantified reliably.

$$Y = \frac{Y_{max} \cdot X}{(K_d + X)} \qquad \textbf{\textit{Equation S1}}$$

Where Y_{max} is the maximum specific binding (100 % DPH fluorescence) and K_d is the binding dissociation constant.

Additional Figures and Tables

Table S1. Sequences of *de novo* designed PNT-forming peptides. The sequence of CC-Hex-T+co differs slightly from CC-Hex-T+; tyrosine (Y) was used instead of W as a chromophore to minimize hydrophobicity and the *C*-terminal E was changed to a *C*-terminal Q to circumvent intramolecular anhydride formation.[1]

Register	***abcdefg abcdefg abcdefg abcdefg***
CC-Hex-T	H-LKAIAQE LKAIAKE LKAIAWE LKAIAQE-OH
CC-Hex-T+	H-LKAIAKE LKAIAKE LKAIAWE LKAIAKE-OH
CC-Hex-T+co	H-CKAIAKE LKAIAYE LKAIAKE LKAIAKQ-SBzl

a) **CC-Hex-T+** (H-LKAIAKE LKAIAKE LKAIAWE LKAIAKE-OH, $C_{145}H_{252}N_{36}O_{37}$, M_{calc}: 3091.8, M_{found} $(M+H^+)$: 3093.7, t_r: 8.9 min).

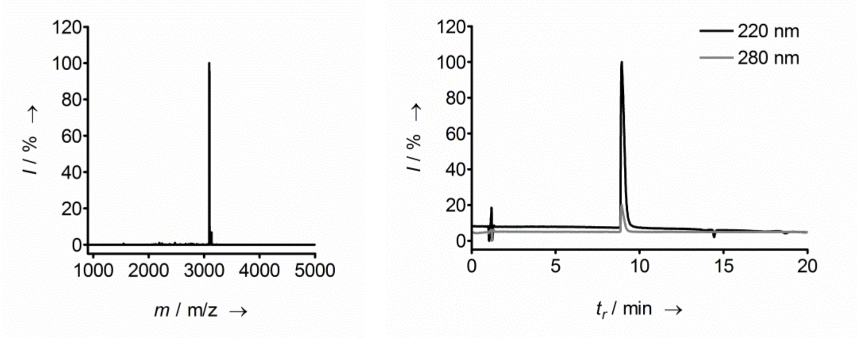

b) **CC-Hex-T+co** (H-CKAIAKE LKAIAYE LKAIAKE LKAIAKQ-SBzl, $C_{147}H_{252}N_{36}O_{36}S_2$, M_{calc}: 3163.9, M_{found} $(M+H^+)$: 3166.0, t_r: 11.2 min).

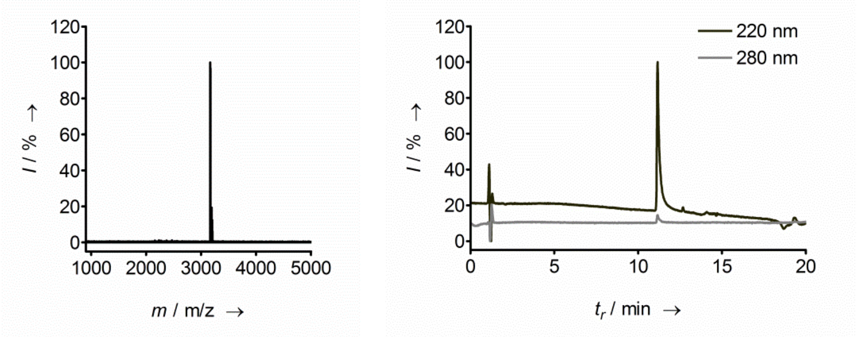

c) **(CC-Hex-T+co)2** reference peptide (Ac-GE LKAIAKE LKAIAYE LKAIAKE LKAIAKQ CKAIAKE LKAIAYE LKAIAKE LKAIAKG-NH$_2$, $C_{289}H_{504}N_{74}O_{76}S_1$, M_{calc}: 6263.6, M_{found} $(M+H^+)$: 6269.8).

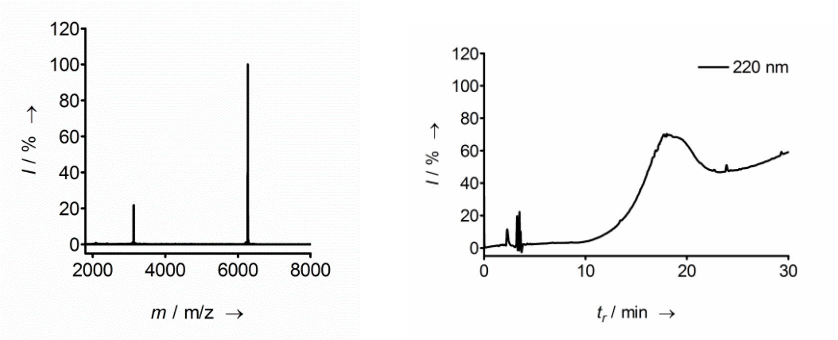

Figure S1. MALDI-TOF spectra and analytical HPLC chromatograms and demonstrating purity and confirming identity of all peptides. MALDI-TOF spectra were recorded in a reflector positive mode. HPLC were run on a reverse-phase C18 column, gradient 20-80% B for 20 min. (CC-Hex-T+co)2 does not resolve on reversed-phase chromatography columns due to aggregation. CC-Hex-T+co was measured on a C4 Nucleosil 120-5 column from Macherey Nagel.

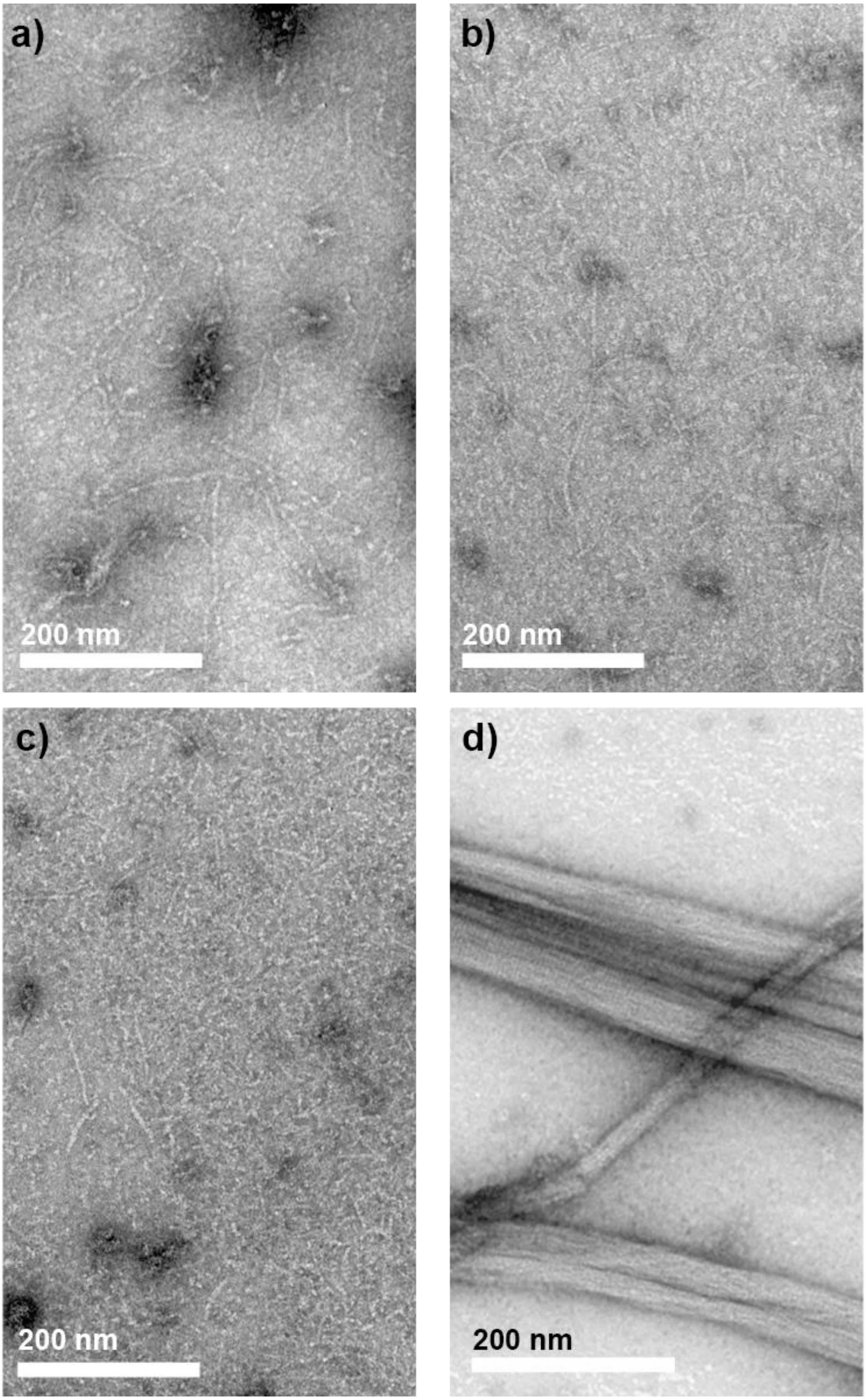

Figure S2. Negative-stain TEM showing the pH sensitivity of CC-Hex-T. a) CC-Hex-T at pH 3.1, b) CC-Hex-T at pH 4.8, c) CC-Hex-T at pH 5.6, d) CC-Hex-T after pH increase from 5.6 to 7.4.[3] The small size of the fibres results in low contrast of the images and therefore increased background noise.

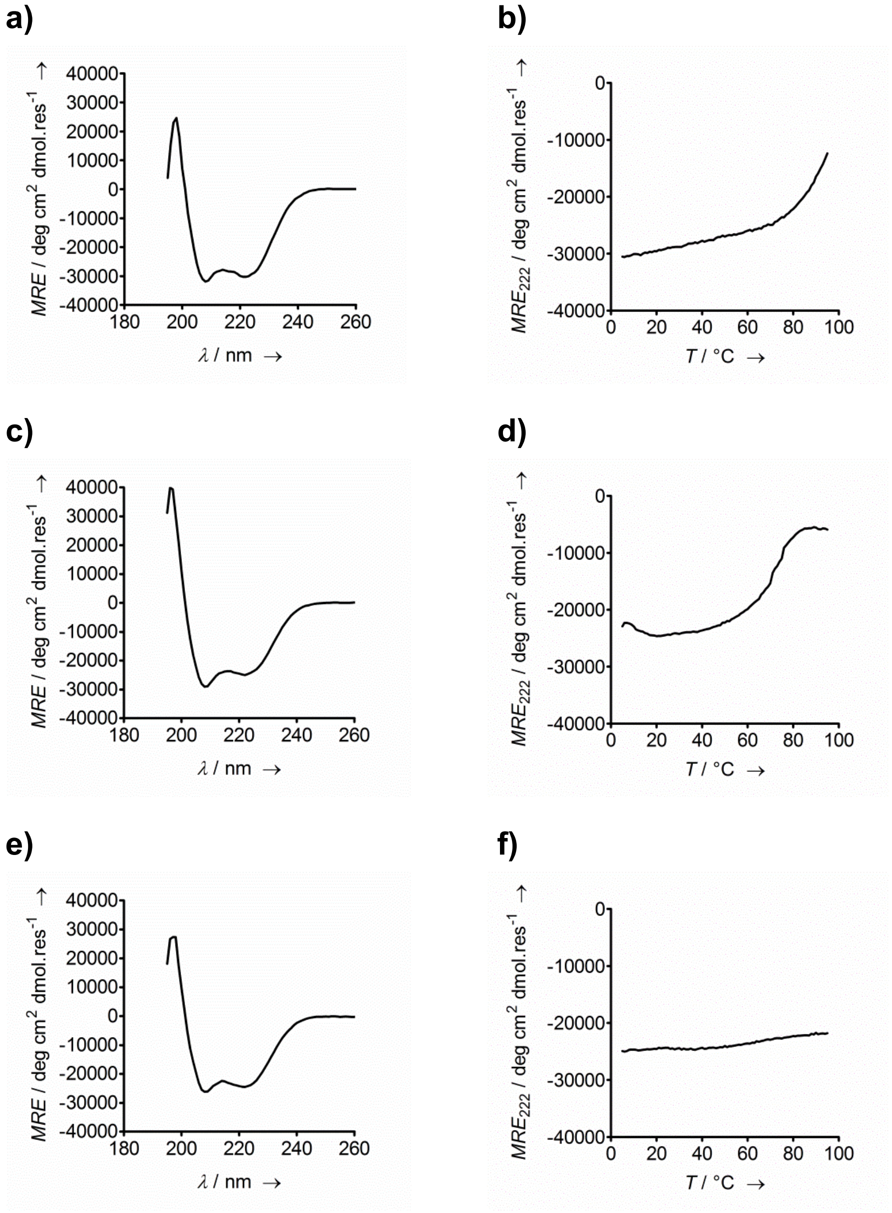

Figure S3. CD spectra and thermal denaturation profiles measured using the CD signal at 222 nm for single PNTs. a, c, e) CD spectra at 20 °C for CC-Hex-T at pH 4.8 (a), CC-Hex-T+ (c), and for CC-Hex-T+co (e). b, d, f) Thermal denaturation curves monitored using the mean residue ellipticity at 222 nm, and plotted as a function of temperature, for CC-Hex-T at pH 4.8 (b), CC-Hex-T+ (d), and CC-Hex-T+co (f). conditions: $c_{peptide}$ = 100 μM.

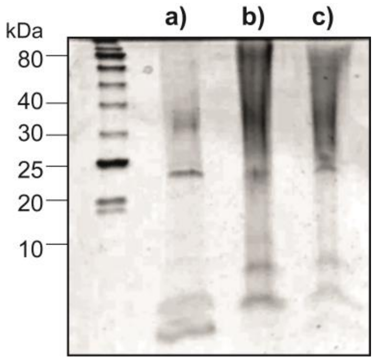

Figure S4. SDS-PAGE analysis of NCL in different buffers after 1 d. a) Denaturing conditions (6 M Gdm·HCl, 100 mM NaH_2PO_4, pH 7.4); b) Phosphate buffer (100 mM NaH_2PO_4, pH 7.4), c) PBS (8.2 mM Na_2HPO_4, 1.8 mM KH_2PO_4, 137 mM NaCl, 2.7 mM KCl, pH 7.4). PNT formation was not successful under a) denaturing condition (formation of small discrete species) and in b) phosphate buffer (poor solubility).

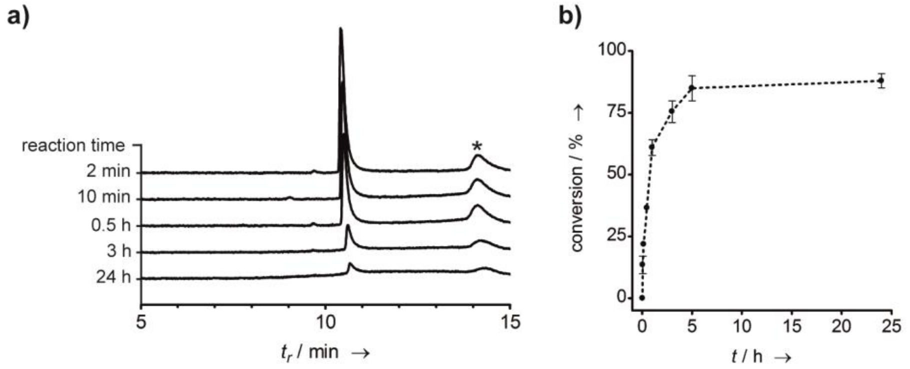

Figure S5. HPLC monitoring of oligomerization of CC-Hex-T+co by NCL. a) HPLC-traces at 220 nm after 2 min, 10 min, 0.5 h, 3 h, and 24 h (gradient: 20-80 % B in 20 min); b) Relative conversion of CC-Hex-T+co over time based on change in peak area in the HPLC chromatograms (* benzyl mercaptane).

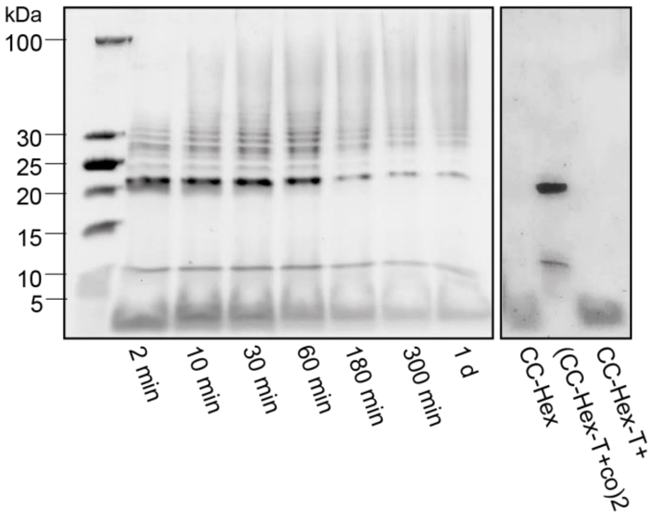

Figure S6. SDS-PAGE of oligomerization reaction sample at different time points and monomeric and dimeric reference peptides. CC-Hex and $(CC\text{-}Hex\text{-}T\text{+}co)_2$ do not self-assemble into fibrous structures because of N- and C-terminal capping. (The GE pattern of the dimeric CC-Hex-T+co could be explained by incomplete denaturation of the peptide in SDS due to the high stability of the assembly. The lower band is in the range of the monomeric 8-heptad CC-Hex-T+co dimer (MW = 6270 Da). We interpret the strong band to be the fully folded peptide. It is possible that due to the high aspect ratio CC-Hex-T+co causes it to have different migration behaviour to the protein standards.)

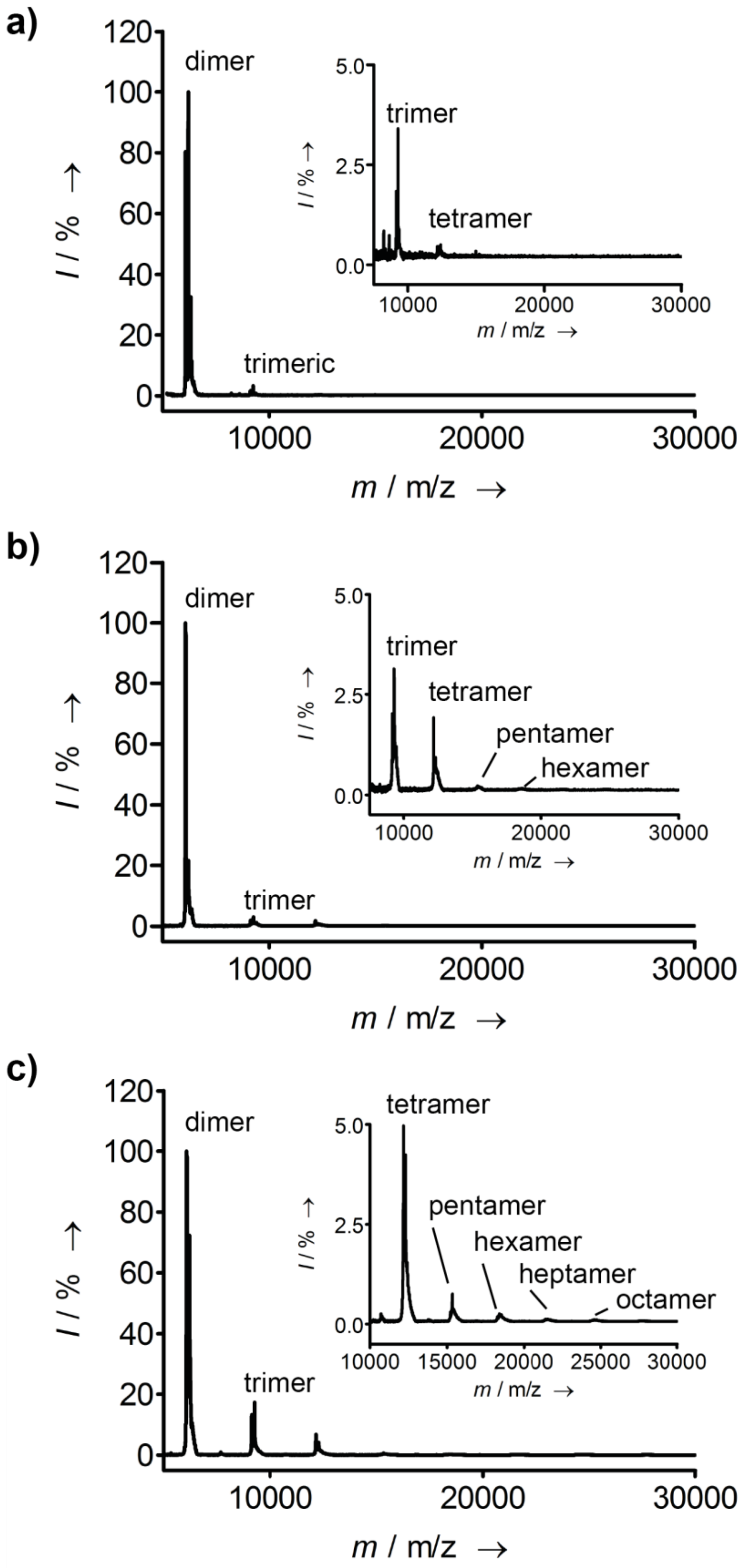

Figure S7. MALDI-TOF mass spectra of the covalent PNT formation with CC-Hex-T+co at a) 10 min reaction time, b) 30 min reaction time, and c) 3 h reaction time. dimer: linear (6205 Da) and cyclic (6081 Da), trimer: linear (9246 Da) and cyclic (9129 Da), tetramer: linear (12289 Da), pentamer: linear (15332 Da), hexamer: linear (18375 Da), heptamer: linear (21418 Da).

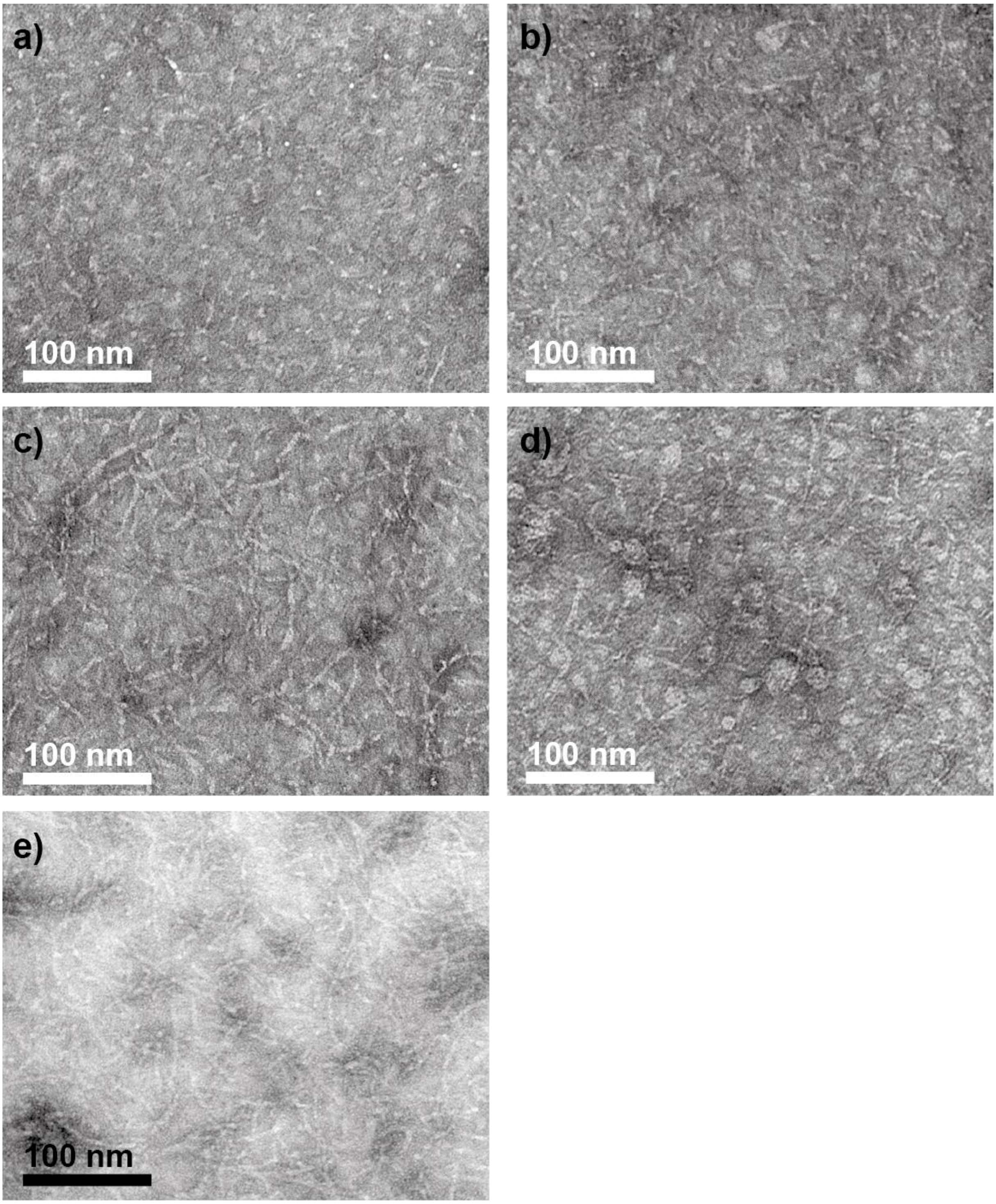

Figure S8. Negative stain TEM at different time points: a) 30 min; b) 60 min; c) 300 min; d) 1 day; and e) 7 days (higher magnification). The small size of the fibres results in low contrast of the images and therefore increased background noise.

a)

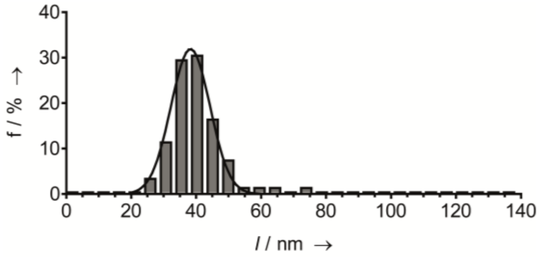

Fibre length: 30 – 45 nm
Sample size: 100 fibres
Normal distribution: $\mu = 38.3$, $\sigma = 6.02$

b)

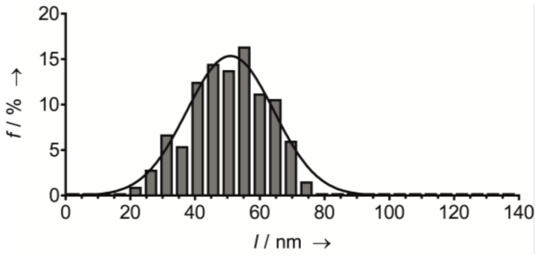

Fibre length: 35 – 65 nm
Sample size: 100 fibres
Normal distribution: $\mu = 50.9$, $\sigma = 13.4$

c)

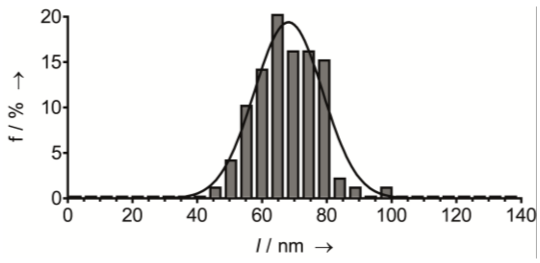

Fibre length: 60 – 80 nm
Sample size: 100 fibres
Normal distribution: $\mu = 68.2$, $\sigma = 10.6$

d)

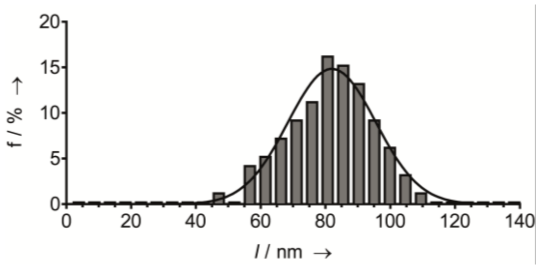

Fibre length: 70 – 95 nm
Sample size: 100 fibres
Normal distribution: $\mu = 82.0$, $\sigma = 13.5$

e)

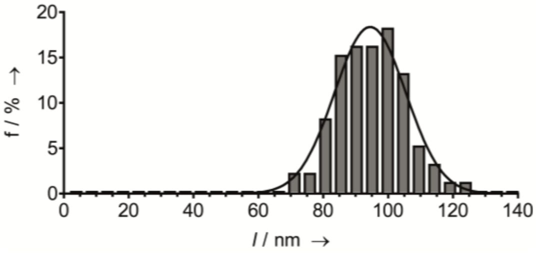

Fibre length: 85 – 105 nm
Sample size: 100 fibres
Normal distribution: $\mu = 94.5$, $\sigma = 11.0$

Figure S9. Distributions of fibre lengths of covalent PNTs measured from negative-stain TEM images.[3] Measurements were made in ImageJ, plotted with bin sizes of 5 nm, and normalized for frequency. Normal probability (black) function fits to the data are given. Fibre samples from oligomerization reaction after a) 30 min, b) 60 min, c) 300 min, d) 1 d, and e) 7 d. Range of fibre lengths were taken from the mean value and standard distribution.

Table S1. Fibre width of PNTs measured from negative-stain TEM images. Measurements were made in ImageJ from 100 fibres as described in the materials and methods section.[3]

**Peptide**	**Fibre width [nm]**
CC-Hex-T, pH 5	3.5 ± 0.5
CC-Hex-T+, pH 7.4	4.0 ± 0.5
CC-Hex-T+co, pH 7,4	4.0 ± 0.5

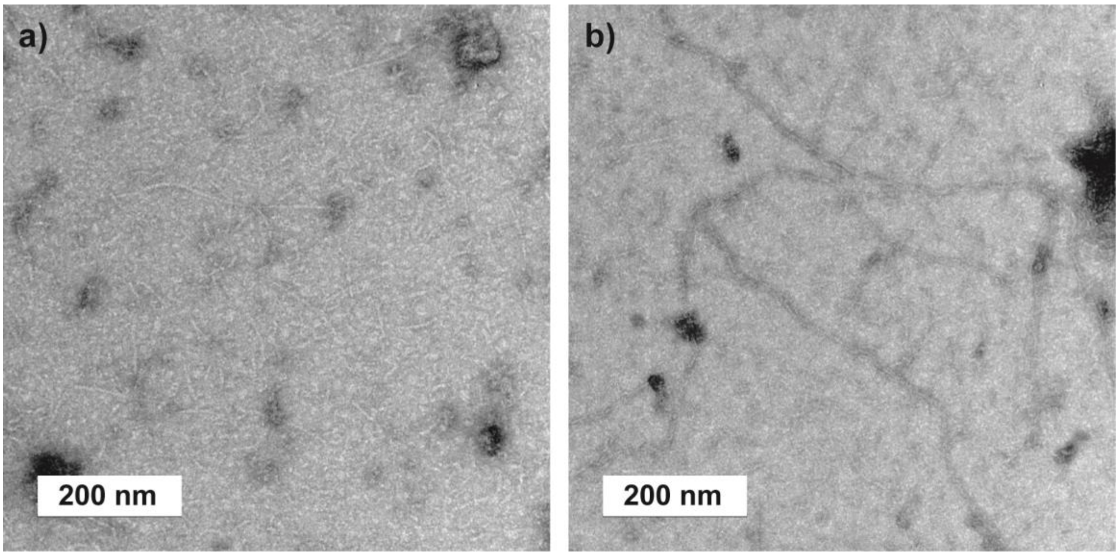

Figure S10. Negative-stain TEM of CC-Hex-T at pH 4.8 a) formed spontaneously and untreated, and b) after heating to 95°C and cooling. The contrast in b) has been increased in order to improve the visibility of the fibres. The small size of the fibres results in low contrast of the images and therefore increased background noise.

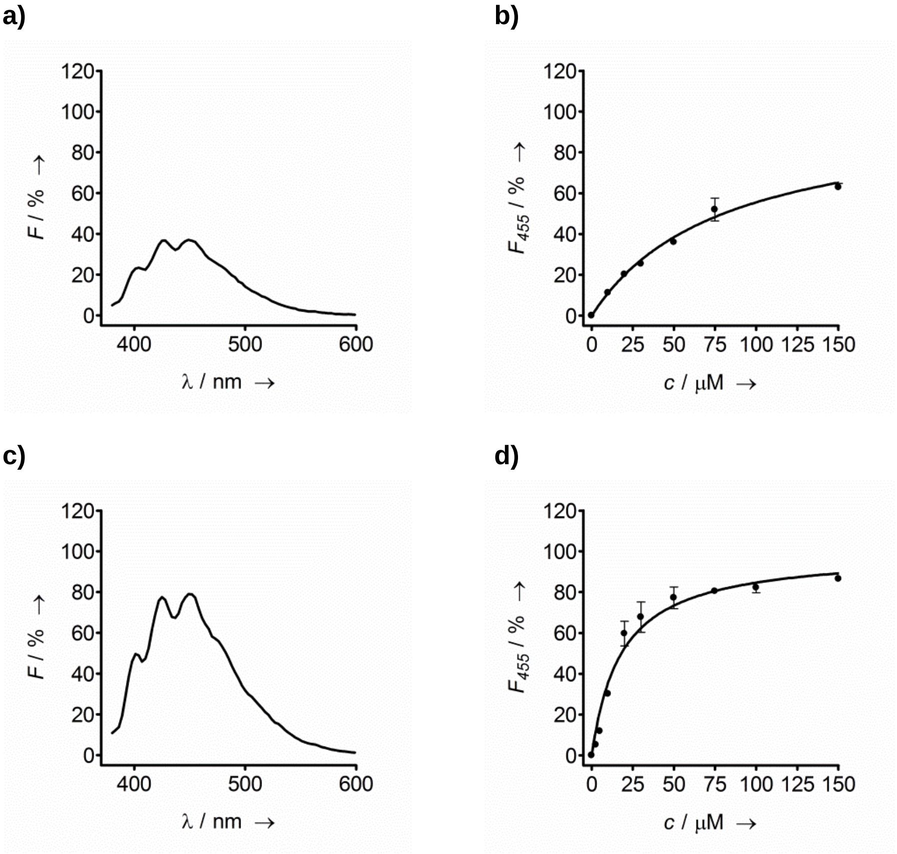

Figure S11. Fluorescence spectra of DPH binding to a) CC-Hex-T+, and c) CC-Hex-T+co. Saturation-binding to DPH of b) CC-Hex-T+, and d) CC-Hex-T+co.

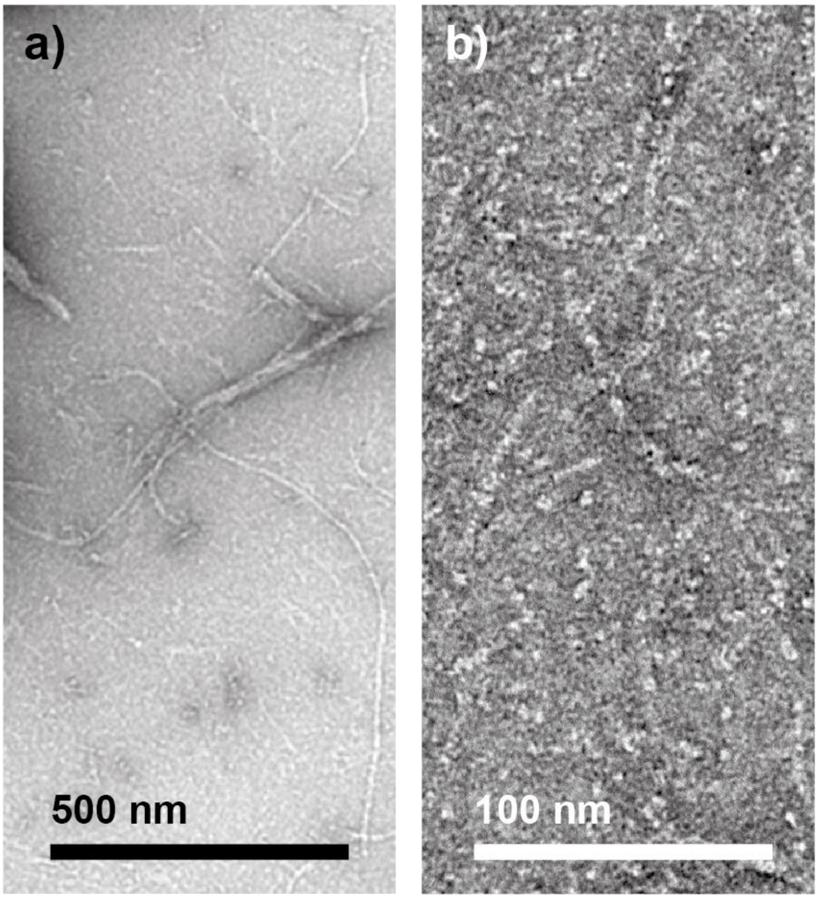

Figure S12. Negative-stain TEM of CC-Hex-T+ 50 mM with 1 mM DPH added a) low magnification, b) high magnification. The small size of the fibres results in low contrast of the images and therefore increased background noise.

References

[1] S. Ficht, R. J. Fayne, R. T. Guy, C. H. Wong, *Chem. Eur. J.* **2008**, *14*, 3620-3629.

[2] H. Rohde, J. Schmalisch, Z. Harpaz, F. Diezmann, O. Seitz, *ChemBioChem* **2011**, *12*, 1396-1400.

[3] C. A. Schneider, W. S. Rasband, K. W. Eliceiri, *Nat. Methods* **2012**, *9*, 671-675.
